# A YOLOv6-Based Improved Fire Detection Approach for Smart City Environments

**DOI:** 10.3390/s23063161

**Published:** 2023-03-16

**Authors:** Saydirasulov Norkobil Saydirasulovich, Akmalbek Abdusalomov, Muhammad Kafeel Jamil, Rashid Nasimov, Dinara Kozhamzharova, Young-Im Cho

**Affiliations:** 1 Department of Computer Engineering, Gachon University, Sujeong-Gu, Seongnam-Si 461-701, Gyeonggi-Do, Republic of Korea; 2 Department of Artificial Intelligence, Tashkent State University of Economics, Tashkent 100066, Uzbekistan; 3 Department of Information System, International Information Technology University, Almaty 050000, Kazakhstan

**Keywords:** fire, flame detection, YOLOv6, deep learning, fire image dataset

## Abstract

Authorities and policymakers in Korea have recently prioritized improving fire prevention and emergency response. Governments seek to enhance community safety for residents by constructing automated fire detection and identification systems. This study examined the efficacy of YOLOv6, a system for object identification running on an NVIDIA GPU platform, to identify fire-related items. Using metrics such as object identification speed, accuracy research, and time-sensitive real-world applications, we analyzed the influence of YOLOv6 on fire detection and identification efforts in Korea. We conducted trials using a fire dataset comprising 4000 photos collected through Google, YouTube, and other resources to evaluate the viability of YOLOv6 in fire recognition and detection tasks. According to the findings, YOLOv6’s object identification performance was 0.98, with a typical recall of 0.96 and a precision of 0.83. The system achieved an MAE of 0.302%. These findings suggest that YOLOv6 is an effective technique for detecting and identifying fire-related items in photos in Korea. Multi-class object recognition using random forests, k-nearest neighbors, support vector, logistic regression, naive Bayes, and XGBoost was performed on the SFSC data to evaluate the system’s capacity to identify fire-related objects. The results demonstrate that for fire-related objects, XGBoost achieved the highest object identification accuracy, with values of 0.717 and 0.767. This was followed by random forest, with values of 0.468 and 0.510. Finally, we tested YOLOv6 in a simulated fire evacuation scenario to gauge its practicality in emergencies. The results show that YOLOv6 can accurately identify fire-related items in real time within a response time of 0.66 s. Therefore, YOLOv6 is a viable option for fire detection and recognition in Korea. The XGBoost classifier provides the highest accuracy when attempting to identify objects, achieving remarkable results. Furthermore, the system accurately identifies fire-related objects while they are being detected in real-time. This makes YOLOv6 an effective tool to use in fire detection and identification initiatives.

## 1. Introduction

Each year, the number of fire-related natural disasters increases, resulting in more human deaths. In addition to human and material losses, fires frequently cause extensive economic harm. Both natural and anthropogenic forces are significant contributors. Factors such as dryness, wind, heat appliances, chemical fires, and cooking are conductive to fire ignition. Accidental fires can start with alarming randomness and rapidly spread out of control. To prevent unforeseen fires and ensure the safety of individuals, prompt evaluation of potential threats and prompt mitigation are necessary. According to the State Fire Agency of Korea, there were 40,030 fires in the country in 2019, which resulted in 284 deaths and 2219 hospitalizations [1]. The number of fires caused record-breaking levels of property damage. Thus, numerous research organizations have implemented tangential techniques for identifying fires. Fire alarm systems, sensor-based frameworks, and other sensing technologies are just a few examples of the warning systems and identification devices that have been adopted over the past several decades to detect specific fire and flame characteristics; however, numerous issues remain unresolved [2]. Recent research has proved the effectiveness of computer vision and deep learning-based methods for fire detection. Computer vision and artificial intelligence (AI) based approaches, such as static and dynamic texture analysis [3,4], neural network convolutions (CNNs), and 360-degree sensors [5,6,7], are widely used in the field of fire detection. We created an autonomous, trustworthy, and robust technique for detecting fires using a novel form of the CNN and the YOLOv6 architecture. This technique was developed in response to the obstacles described above. This research aimed to identify signs of an unexpected fire in order to protect lives and property. Flames are characterized by a variety of sizes, colors, movements, shapes, speeds, and appearances, as well as any combination of these characteristics. Even though these factors make fire detection more challenging, we feel there is a significant chance that technologies can be developed for automatic application. The following are some of the most important advantages of the proposed strategy:

i. We will publish online a significant dataset for use in fire detection, including examples of daytime and nighttime fire and flame scenarios. A deep CNN learns key information from enormous databases in order to make accurate predictions and minimize overfitting.
ii. We provide a YOLOv6-based active fire detection method to bolster defenses and avoid a lengthy operation.
iii. While rotating fire datasets by 15 degrees, a mechanism was devised to mechanically reorder flagged containers.
iv. During the training phase of YOLOv6, class predictions were generated utilizing independent logistic classifications and a binary cross-entropy loss. This is far faster than other detecting networks.
v. We used fire-like images and eliminated low-resolution images to reduce the number of false positives in the fire identification technique. In addition, the proposed model significantly increases the precision and decreases the false detection rate, even in small fire regions.

The primary objective of YOLO’s most recent version, YOLOv6, is improved object detection accuracy, particularly for recognizing small objects. YOLOv6 is an innovative system for real-time object detection based on deep learning. YOLOv6 is superior for object recognition because of its exceptional speed and accuracy. It outperforms other cutting-edge object detection algorithms in terms of speed, accuracy, and power consumption. This study employed YOLOv6, a computationally efficient deep learning system, to locate and characterize fires. YOLOv6 increased the detection and recognition of fires in moving and still photos with an accuracy of over 90%. One of the datasets used to train the model includes roughly 3000 photographs of fires. For the test phase, YOLOv6 received a score of 0.9 F1, demonstrating its successful detection ability and identification of fire. This study was conducted to evaluate YOLOv6’s usefulness as a fire detection tool. To evaluate YOLOv6’s use in fire recognition and detection in Korea, we conducted tests and simulations. The results proved YOLOv6’s applicability for fire detection by proving its capacity to detect active flames while lowering the number of false positives by a large margin. YOLOv6 is a cutting-edge deep learning algorithm for object detection, and is trained using images from Google’s image search, YouTube videos, and other sources. This study focused on the application of YOLOv6 for fire detection and evaluated its efficacy.

This paper’s remaining sections are organized as follows: The second section reviews the research on classical and deep learning techniques for identifying specific fire zones. In Section 3, the suggested fire detection method is detailed. In Section 4, we discuss the results of quantitative and qualitative tests, as well as our dataset. Some of the drawbacks of the suggested method are described in Section 5. Section 6 finishes the paper with a review of our findings and recommendations for further research.

## 2. Related Work

The field of image recognition has witnessed the rise of a particular type of deep neural network (DNN), CNN. Learnable neural networks comprise numerous layers, each of which performs a separate function when extracting or identifying features. Computer vision, a compelling form of AI, is ubiquitous, and is often experienced without us realizing it. Image processing is the area of computer vision and science devoted to imitating elements of the human visual system and enabling computers to discern and process objects in images and videos similarly to humans.

### 2.1. Fire Detection Strategies Based on Image Processing and Computer Vision

Location, rate of spread, length, or surface are only a few of the geometrical features of flames that Toulouse et al. [8] aimed to identify with their novel approach. The pixels depicting the fire were sorted into categories based on their color nonrefractive pixels were able to detect smoke sorted based on their average intensity. The edge computing framework for early fire detectors developed by Avgeris et al. [9] is a multi-step process that significantly facilitates border identification. However, these computer-vision-based frameworks were only used on relatively static images of the fire. Recently developed techniques based on fast Fourier transform (FFT) and wave variation have been utilized by other researchers to analyze the boundaries of wildfires in movies [10]. Studies have demonstrated that these methods are effective only in specific scenarios.

Color pixel statistics have been used to examine both foreground and background photos to search for signs of fire. By fusing color information and recording foreground and background frames, Turgay [11] created a real-time fire detection system. Fire color data is derived from statistical assessments of representative fire photos. The pixel value color information in each color channel is modeled using three Gaussian filters. This technique is used for simple adaptive data scenarios. Despite the widespread use of color in flame and fume recognition, such methods are currently infeasible due to the influence of environmental factors such as lighting conditions, shadows, and other distractions. Even though the fire has long-term dynamic movements, color-based approaches are inferior to the new dynamics for fire and smoke detection.

By analyzing the motion of smoke and flames with linear dynamic systems, researchers in [3] created a method for detecting fires (LDSs). They discovered that by including color, motion, and spatial-temporal features in their model, they could achieve both high detection rates and a considerable reduction in false alarms. We aim to enhance the efficiency of the current fire detection monitoring system, which issues early warning alerts, by employing two different support vector classifier methodologies. To locate forest fires, researchers analyzed the fire’s spatial and temporal dynamic textures [12]. In a static texture investigation, hybrid surface descriptors were employed to generate a significant feature vector that could differentiate flames or distortions from each other without using conventional texture descriptors. These approaches rely heavily on easily discernible data, such as the presence of flames in images. The appearance of fire is affected by several factors, including its color, movement speed, surroundings, size, and borders. Challenges to using such methods include a poor picture and video quality, adverse weather, and an overcast sky. Therefore, modern supplemental methods must be implemented to enhance current methods.

### 2.2. Techniques for Fire Detection Based on Deep Learning Approaches

Recently, several deep learning (DL) techniques have been effectively applied in various fields of fire and face detection research [13,14,15]. In contrast to the manual qualities of the techniques we have studied, DL methods can automate the selection and removal of features. Automatic feature extraction based on learned data is another area where DNNs have proven useful [16,17]. Rather than spending time manually extracting functions, developers may instead focus on building a solid dataset and a well-designed neural network.

We have previously presented [4] a novel DL-based technique for fire detection that uses a CNN with dilated convolutions. To evaluate the efficacy of our approach, we trained and tested it using a dataset we created, which contained photographs of fire that we collected from the web and manually tagged. The proposed methodology is contactless and applicable to previously unseen data. Therefore, it can generalize well and eliminate false positives. Our contributions to the suggested fire detection approach are fourfold: this proposal includes a custom-built dataset, a few layers, small kernel sizes, and dilation filters all used in our experiments. Researchers can find this collection to be a valuable resource for utilizing images of fires and smoke.

To improve feature representations for visual classifications, Ba et al. [2] created a novel CNN model called Smoke Net that uses spatial and flow attention in CNN. An approach for identifying flames was proposed by Luo et al. [18], which uses a CNN and smoke’s kinetic characteristics. Initially, they separated the potential candidates into two groups, one using the dynamic frame references from the backdrop and the other from the foreground. A CNN with five convolutional layers plus three fully linked layers then automatically retrieved the candidate pixels’ highlights. Deep convolutional segmentation networks have been developed for analyzing fire emergency scenes, specifically for identifying and classifying items in an image based on their construction information regarding color, a relatively brilliant intensity compared to its surroundings, numerous shifts in form and size, and the items’ propensity to catch fire [19].

The proposed CNN models enabled a unique picture fire detection system in [20] to achieve maximum accuracy of 83.7%. In addition, a CNN technique was utilized to improve the performance of image fire detection software [21,22,23,24]. Algorithms based on DL require large amounts of information for training, verifying, and testing. Furthermore, CNNs are prone to spurious regressions and are computationally expensive due to the large datasets required for training. We compiled a large dataset to address these issues, and the associated image collections will soon be made accessible to the public.

### 2.3. Fire Detection Approaches Based on YOLO (You Only Look Once) Networks

YOLO, invented in 2016 by Joseph Redmon et al. [25], is an object detection system. Built on CNNs, it was developed to be quick, precise, and adaptable. This system comprises a grid of cells that divide an image into regions, a collection of bounding boxes that are employed to detect objects within those regions, and a collection of predefined classes that are associated with those regions. The YOLO system takes an input image and divides it into a grid of cells, with each cell representing a different area of the image. Thereafter, the system analyzes the regions and places them into one of several categories based on the types of objects found there. Once an object has been recognized, the system constructs a bounding box within it and assigns a class to the box. With the object’s identity established, the system can calculate its coordinates, dimensions, and orientation. Park et al. suggested a fire detection approach for urban areas that uses static ELASTIC-YOLOv3 at night [26]. They recommended using ELASTIC-YOLOv3, which is an improvement on YOLOv2 (which is only effective for detecting tiny objects) and can boost detection performance without adding more parameters at the initial stage of the algorithm. They proposed a method of constructing a movable fire tube that considered the particularities of the flame. However, traditional nocturnal fire flame recognition algorithms have these issues: a lack of color information, a relatively high brightness intensity compared to the surroundings, different changes in shape and size of the flames due to light blur, and movements in all directions. Improved real-time fire warning systems based on advanced technologies and YOLO versions (v3, v4, and v5) for early fire detection approaches have been previously proposed [27,28,29,30].

## 3. Proposed Work

### 3.1. Fire Dataset Description

**Figure 1:**
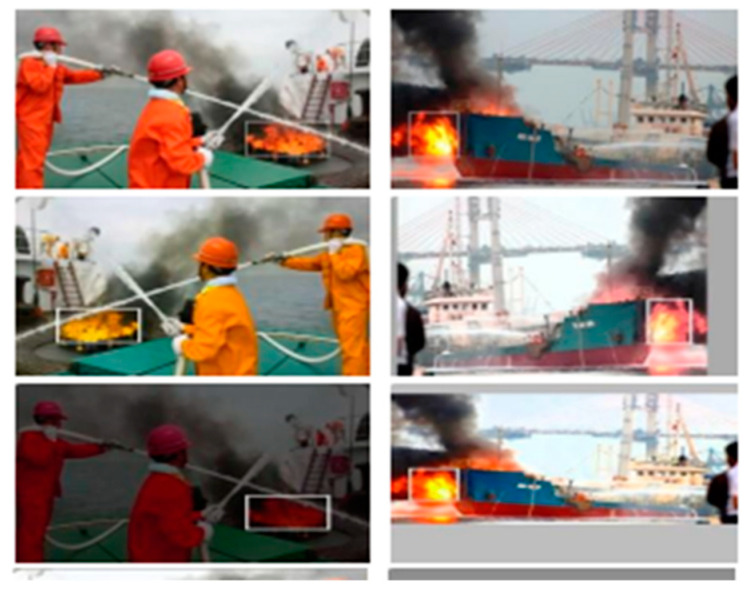
Example fire dataset images.

Images of fires from various sources were utilized to train the YOLOv6 model. Images captured at various focal lengths and under a range of lighting situations are included in the dataset. We searched the Internet for videos of fire scenes and captured 4000 frames of those videos, as shown in Table 1. They were divided as follows: 70% for training, 20% for testing, and 10% for validation. Additionally, there are three sizes of fire images in the datasets: small, medium, and large. This illustration displays typical fires, featuring flames and burning items. There are images of enormous fires with burning automobiles and buildings. Various transformations, including scaling, rotation, and flipping, are applied to both small and medium fire pictures to improve them. To train the YOLOv6 model in fire detection, the dataset offers a valuable and diverse collection of fire images. The dataset’s diversity strengthens the model’s ability to generalize to unseen or unexpected data and adapt to changing conditions. Altogether, we collected 4000 images of fires, both diurnal and nocturnal, for our training dataset (Figure 1).

After collecting the data, the dataset was still found to be small. To achieve more accurate results, we used publicly available robmarkcole and Glenn–Jocher databases, as shown in Table 2 [31,32]. Overfitting may arise because of several factors, including a dearth of training data and insufficient data points to adequately capture all conceivable input values. One of the most effective approaches to overcome overfitting is increasing the training dataset using data augmentation techniques. We found that image data augmentation approaches, that is, geometric (affine) transformations, brightness/contrast enhancement, and data normalization, were the most effective in increasing the number of dataset images and improving the final accuracy rate, as demonstrated by experiments [33,34]. The effectiveness of DL models depends on the size and resolution of the training image datasets. Hence, it is important to extend the training dataset by data augmentation. Therefore, we rotated each original image and then flipped each rotated image horizontally to increase the number of images in the fire images dataset. By applying the data augmentation methods to the original fire images, we increased the total number of images significantly.

We rotated each image by 15 and 45 degrees, thereby increasing the number of photos in the collection. We doubled the number of images we started with using a technique called dataset augmentation, which involves making new, slightly different versions of the samples already included in the training set. There are currently over 11,000 datasets after enhancement. Table 3 further illustrates that over 2300 fire-related photographs were utilized to further minimize the number of false positives. To maximize the performance of fire detection, it is necessary to use fire-like images in the training datasets, as detailed in our previous published papers [27,28,29,30]. The dataset we utilized has three folders: train, test, and val. The entire fire picture dataset was divided into training (70%), test (20%), and val (10) sets. To train, test, and validate the model, each folder includes a set of both labeled and unlabeled photos. Moreover, as we do not have wildfires, we used YouTube videos of various fire forms and types to evaluate our model and ensure its correctness [35].

**Figure 2:**
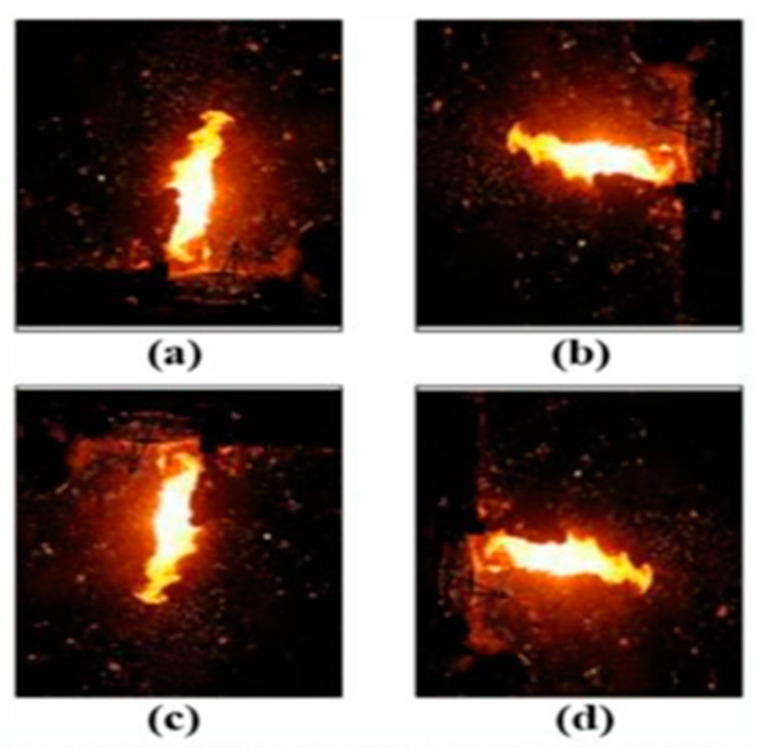
Original image (**a**); 90° rotation image (**b**); 180° rotation image (**c**); 270° rotation image (**d**).

Initially, we rotated all the fire images by 90, 180, and 270 degrees (Figure 2). The results did not drastically shift when we rotated the fire images by over 15 degrees. Contrarily, we might lose the fire image’s region of interest (ROI) when we rotate them by over 15 degrees [36].

### 3.2. Methodology

The YOLOv6 detection model is an improvement over its predecessors, YOLO and YOLOv1–5. The YOLOv6 CNN is a single-stage detection algorithm, meaning it can identify objects in an image without first passing them through a regional proposition network (RPN). This accounts for the speed and precision of detection and shrinks the parameters of the model. The recall rate of older YOLO algorithms, such as YOLOv1, is inferior to that of regional proposal-based systems, and their location is also less precise. YOLOv2 leverages batch normalization, an established technique, and multi-scale training to improve its responsiveness to inputs of varying sizes while preserving speed and accuracy. Despite its improved accuracy, YOLOv2 has yet to meet the requirements for widespread industrial use. A few tweaks to softmax loss and some complex use of logistic regression per category led to YOLOv3’s enhancements. There is potential to further improve YOLOv3’s objective. YOLOv4 is a faster version of the software used on the dark web and in PyCharm for object detection, but it is still overly slow to be practical. A development of the YOLOv1–YOLOv4 network, the YOLOv5 network consists of three architectures: the head, which generates YOLO layers for multi-scale prediction; the neck, which improves information flow based on the path aggregation network (PANet); and the backbone, which is based on a cross-stage partial (CSP) integrated into the Darknet [37,38]. Before being sent to PANet for feature fusion, the data were sent to CSPDarknet for feature extraction.

**Figure 3:**
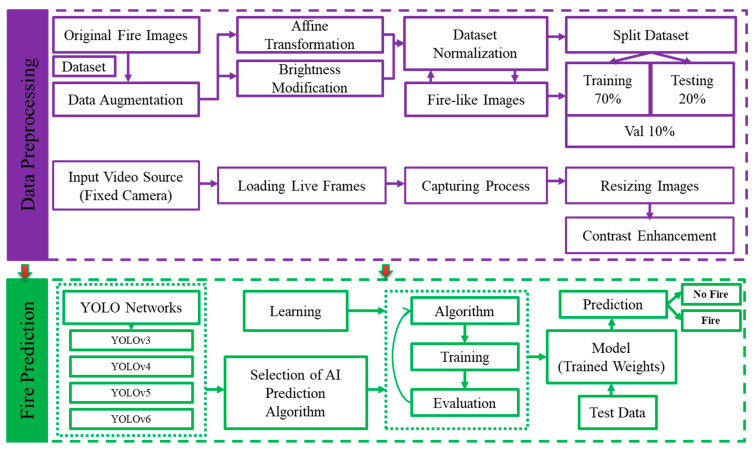
Flowchart of the proposed fire detection and classification approach.

We divided the fire detection and classification procedure into two parts, as shown in Figure 3. The section on data processing describes dataset gathering and enhancement using various data augmentation techniques. The selection of a suitable YOLO model to use for fire detection is the main goal of the fire prediction stage. We decided to utilize the YOLOv6 network for the efficient identification and warning of fire disasters after training and testing the default YOLO approaches in fire detection instances.

YOLOv6 is an updated version of YOLO designed to enhance the precision with which small objects may be identified. Single-channel improved YOLO (SEC-YOLO) and multi-channel enhanced YOLO (MEC-YOLO) are its two main parts (MCE-YOLO). SEC-YOLO employs a single-channel method for detecting subtle phenomena. This structure is made up of 32 convolutions and four linked network layers. To recognize tiny objects, MCE-YOLO employs a multi-channel technique that uses three convolution operations and five linked network layers. The YOLOv6 model combines the single- and multi-channel techniques to achieve more accurate object detection than previous YOLO versions [39].

**Figure 4:**
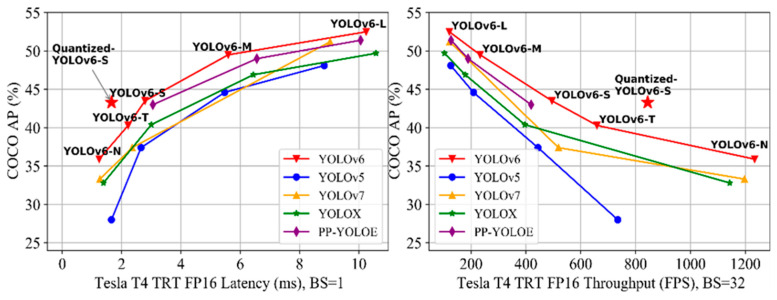
YOLOv6 accuracy comparisons with other YOLOv detectors [41].

There are multiple model sizes available in YOLOv6 (N, T, S, M, and L) to optimize the performance trade-off depending on the scenario’s industrial application. Furthermore, some bag-of-freebies techniques, such as identity and additional training epochs, are introduced to further enhance performance. To achieve maximum efficiency in an industrial setting, we use QAT in conjunction with channel-wise distillation and graph optimization. YOLOv6-N achieved 35.9% AP on the COCO dataset with 1234 FPS on T4, while YOLOv6-S and its quantized version achieved 43.5% AP and 43.3% AP at 495 FPS and 869 FPS, respectively, on T4. Similar to YOLOv6, YOLOv6-T/M/L exhibits maximum performance, with superior accuracy compared to other detectors of comparable inference speed, as shown in Figure 4 [40].

### 3.3. Fire Detection Using YOLOv6

The YOLOv6 model only has a single detection step. The following is a detailed account of how fires may be detected using YOLOv6. If the target to be identified lies within an S 2 grid, that grid is responsible for determining the object to be detected. The network begins by dividing the input fire picture into S grids. Following this, one grid provides predictions for three bounding boxes, along with confidence values for these predictions. In this context, “confidence calculation” means:

Confidence = Pγ × IoUpred truth, Pγ(object) ∈ [0, 1](1)

where P = 1 if and only if the target is inside the grid; otherwise, P = 0. The IoU indicates how well the actual bounding box matches the projected one. The degree of certainty indicates both the presence or absence of items in the grid and the precision with which their bounding boxes can be predicted. Non-maximum suppression (NMS) is the mechanism used by the YOLO network to select the optimal bounding box when many boxes detect the same targets simultaneously.

**Figure 5:**
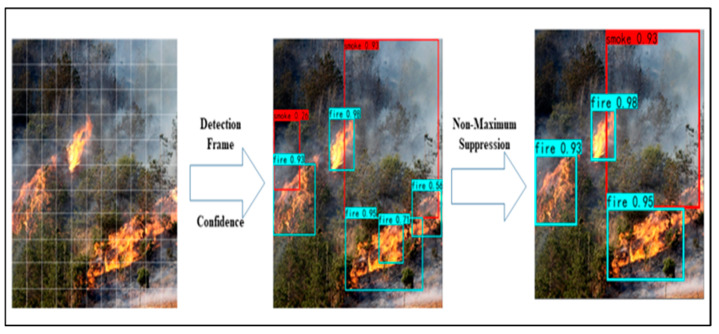
Input image is divided into S × S grids, and each grid predicts three bounding boxes and confidence scores. Afterward, the optimal bounding box is selected using an NMS method.

CNNs can accurately categorize flames and smoke in films, making them useful for fire detection [42]. To aid in the detection of minute flame areas, the process of Ref. [43] enhances the YOLOv6 model by including several feature balances with finer resolution. The YOLOv6 model suggested in this study achieves a more symmetrical network structure by taking into account depth, breadth, and resolution, as previously proposed [43,44,45]. Detection of a YOLOv6 fire may be broken down into processes, as depicted in Figure 5.

Each of the S grids containing the input image makes predictions for three bounding boxes and confidence levels. Thereafter, a non-maximum suppression technique is used to select the best possible bounding box. The Efficient Net proposal [46] provides an opportunity to develop a standardized approach to scaling CNNs. Network depth, breadth, and resolution may be better matched without requiring extensive human adjustment, leading to a more well-rounded network design, as shown on the fourth of fourteen pages in the volume entitled “Sustainability: 2022”. The simple and efficient composites scaling technique may further increase the accuracy of fire detection in comparison to existing single-dimensional scale methods, and can conserve computational resources because a major proportion of detection objects inside the fire detecting dataset are small flames. There is no question that the Efficient Net utilized in this article has distinct advantages over CNN, including faster processing, reduced model size, and fewer parameters. Figure 2 depicts the proposed network architecture of the YOLOv6 fire detection model. The fire-feature YOLO’s extraction network employs a mobile inverted bottlenecks convolution (MBConv) [47], which is made up of depth-wise convolution and compression excitation networks (SENets) [48], as opposed to the residual block employed by the extracting features network, Darknet53, in YOLOv6. The MBConv block first uses a 1×1 convolution tone to up-sample the image, then passes the up-sampled image through depth-wise convolution and SENet in turn, and finally down-samples the picture using a 1 1 convolution kernel. Up-sampling and feature extraction on the input picture yields the vector feature matrix known as the matching feature map.

A convolutional set made up of five convolution layers with alternating 1 × 1 and 3 × 3 kernels processes the feature map to extract higher-level features extracted from a tiny target. Once the activation function is applied, the accumulation layer is utilized to transform the high-dimensionality between one vector. The most likely classification outcome is then chosen as the extracting features network output after these vectors are fed into the activation function. Targeting the feature extraction network’s inability to strike a good balance between depth, width, and resolution, this paper proposes using the enhanced Efficient Net extracting features network only on the fire dataset to remedy the former’s shortcomings in tiny target detection and boost the latter’s feature extraction capacity. The capacity to learn minute target characteristics is augmented and the effectiveness of a network for extracting features from small targets is improved, thus enabling the processing of small target images.

## 4. Experimental Results and Discussion

To validate the algorithm’s effectiveness, this study conducted numerous tests on the test images using the trained YOLOv6 model. The accuracy, recall, F1, and AP values are the key indices for gauging the accuracy of a neural network model. Samples in the binary classification issue can be classified as true positives (TP), false positives (FP), true negatives (TN), or false negatives (FN), depending on the relationship between the actual and expected categories (FN). The confusion matrix of the categorization is displayed in Table 4.

The F-measure (FM), which balances the precision and recall rates and measures the weighted average, was tested. This rating considers both the true positive and false negative rates. The FM is the characteristic that detects an object most frequently because it is challenging to measure the accuracy rate. False negatives and true positives performed better in a detection model that used the same weight. Precision and recall, however, must be considered if real positives and false negatives are different. The ratio of genuine positive observations is known as precision.

As described in earlier studies [49,50,51,52,53,54,55,56], recall is a false positive observation ratio in contrast. Our suggested model achieved a 98.3% accuracy rate and a 1.7% false detection rate. The average precision and recall rates of our suggested method can be calculated as illustrated in Equations (2) and (3):

(2) $$Precision=\frac{TP}{TP+FP},$$

(3) $$Recall=\frac{TP}{TP+FN},$$

When the accuracy ratio is plotted against the recall rate, the resulting graph is called a precision–recall curve (P-R plot). The effectiveness of the model may also be determined by its FM score.

The FM score can be defined as follows:

(4) $$FM=\frac{2\times precision\times recall}{precision+recall}$$

The average accuracy of each detection was also employed as a criterion in this investigation (AP). The following is a definition of the term:

AP = Precision (Recall)*d*(Recall) 1

**Figure 6:**
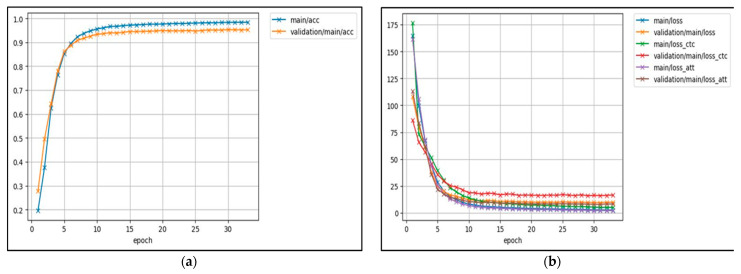
The training processes of the proposed method: (**a**) accuracy vs. epoch and (**b**) validation loss vs. epoch during network training.

Figure 6 show the training accuracy and training loss for the model. Loss is a value that represents the summation of errors in our model. Errors occurred mainly because of the casting of the appropriate sign presence of fire-like sunset and sunrise situations, fire-colored objects, and architectural lighting. Accuracy measures how well our model predicts by comparing the model predictions with the true values in terms of percentage. Having a low accuracy but a high loss would mean that the model makes big errors in most of the data. However, if both loss and accuracy are low, it means the model makes small errors in most of the data. However, if they are both high, it makes big errors in some of the data. Finally, if the accuracy is high and the loss is low, then the model makes small errors for just some of the data, which would be the ideal case [57].

### 4.1. Analysis by Experiment

This section provides an overview of the experimental setup, dataset, model assessment indicators, and experimental results analysis of the training network. The superiority of the novel model provided in this research was examined through various comparison tests on different models. Experiments were conducted using a fire dataset and a small target dataset. Verifying the detection on the small object fire dataset ensures the precision of the target tracking network and provides an evaluation of its performance in complex environments. These environments include, for example, the detection of targets under varying lighting conditions and smoke that resembles a fire. The findings verified that smaller targets are more easily detected with YOLO. Because GPU performance is limited, the sampling size is fixed to 8, each model trains 100 epochs, and the learning basics rate is 103, which is divided into 10 after 50 epochs for the YOLOv6 detection model.

We implemented and tested the proposed configuration from the Anaconda 2020 Python distribution on a computer with an 8-core 3.70 GHz CPU, 32 GB RAM, and NVidia GeForce 1080Ti GPU, as indicated in Table 5.

Hardware: The central processing unit (CPU) is an option for running YOLOv6, albeit it is much slower than using a graphics processing unit (GPU). A recent multi-core CPU should be sufficient with small datasets or models.

Graphics processing unit (GPU): YOLOv6 is GPU-compatible. For optimal performance, an NVIDIA graphics processing unit (GPU) with CUDA support and 8 GB of RAM is required. How quickly inference can be performed is a function of the two different GPU architectures and the number of CUDA cores.

Random Access Memory (RAM): RAM is needed for handling the dataset or model. While 8 GB of RAM is sufficient for most applications, 16 GB may be needed for larger datasets and models. The dataset, YOLOv6 models, and other files needed will take up storage on the machine. The amount of space needed to store the dataset and model will vary.

### 4.2. Detection Performance of Small Targets

Because the lens is far from the fireplace, only a tiny portion of the collected image will contain the fire, making it difficult for the network model to identify the presence of flames and smoke. A comparison of three target detection methods on the tiny target firing dataset shows that the proposed YOLOv6 outperforms the test Faster R-CNN and the unimproved YOLOv3 network in terms of detection efficiency for extremely small target objects. To improve the interplay between pieces of data, YOLOv6 trained only on fire small object data performed adaptive alterations in depth, breadth, and resolution for tiny target pictures to be identified. Thereby, YOLOv6’s feature extraction for tiny targets is enhanced, and the precision with which small targets may be detected is increased. The results of the evaluation of the three modeling techniques on a small target firing dataset are presented in Table 6.

Our custom dataset includes 370 photos that we captured ourselves. The dataset only contains images of smoke and flames with relatively tiny targets. An extremely small region of the image contains the recognized target when a fire image of size 250 × 250 is embedded within an image of size 1850 × 1850.

Comparing the three models’ performance on the small object firing dataset reveals strikingly varied detection efficiencies. Our proposed YOLOv6 model outperforms competing models in terms of both accuracy and recall rate. Small targets may be detected with an accuracy of at most 93.48 percent. When detected early, forest fires cause less damage to the ecosystem, incur fewer losses, and have a reduced risk of spreading, all of which promote sustainable growth.

**Figure 7:**
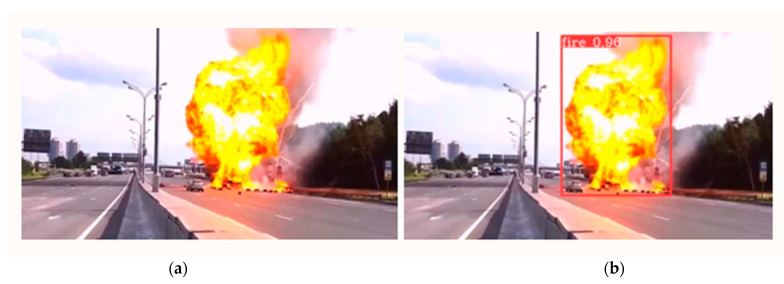
Fire scenarios included experiments: (**a**) input image and (**b**) output image with fire detected region.

The model trained with YOLOv6 networks exhibits high efficacy in determining firing targets. We employed the YOLOv6 model to detect minuscule targets of fire and graphically present the findings. The small target fire dataset only contains images from the validation dataset, which are the targets of the firing. Figure 7 displays the final image detection results after YOLOv6 was validated in over 30 verification examples. In comparison to YOLOv3 and Faster R-CNN, whose detection results only show partial targets, YOLOv6 can identify all of the fires and smoke present in the image.

### 4.3. The Model Detection Efficiency in Varying Ambient Lighting

**Figure 8:**
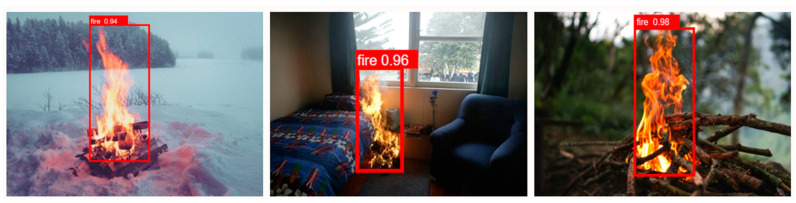
Results of fire detection system for day images.

**Figure 9:**
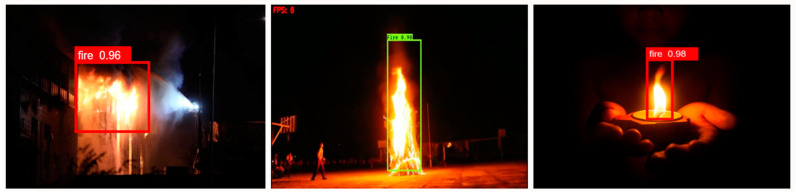
Results of fire detection system for night images.

Here, we tested YOLOv6 in the real world by comparing many images of the fire taken under varying lighting conditions. There will either be limited lighting or a lot of lighting at the site where the fire detection is taking place. This will affect how well fires are detected in this sort of setting. The model’s ability to recognize faint targets is enhanced by a large-scale feature map [58], although it still suffers from over- or underestimation in low-light settings. Figure 8 and Figure 9 can be examined to observe the results of the detection procedure. By equating the model’s detection performance to that of the Faster R-CNN, YOLOv3, and YOLOv6 models, we find that it has excellent performance over a wide range of lighting situations and is highly resistant to variations in illumination. The YOLOv6 model’s benefits can mitigate the destruction caused by forest fires, lessen the effects of global warming on people, and encourage long-term growth in a sustainable direction.

### 4.4. Discussion

**Figure 10:**
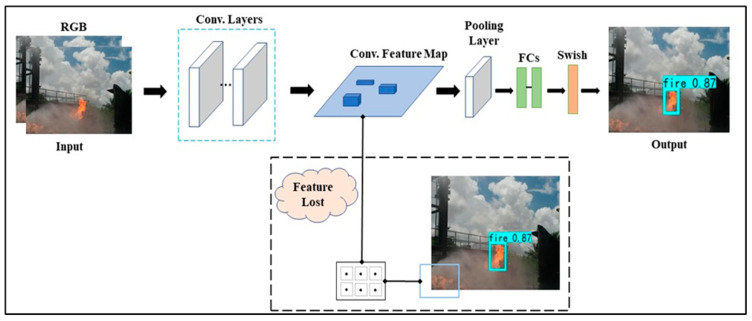
When the YOLOv6 model detects the concealed flame target, it is easy to lose features during model training, resulting in inaccurate detection results.

Here, we analyze YOLOv6’s functionality in various real-world settings. Small target detection, fire and smoke detection, and fire detection under varying light levels have all exhibited promising results when utilizing the YOLOv6 model proposed in this study. In addition to being able to perform real-time detection, it is also resilient in real-world situations. When we tested the method, however, we discovered that it still suffers from the common issues of poor detection accuracy and difficulty in recognizing semi-occluded objects. Figure 10 depicts the conundrum that arises during fire inspections because of the inherent uncertainty in detecting fires in their natural environments. The existing target detection approach also has to address this pressing issue [59]. The good news is that none of these obstacles are insurmountable. The detection algorithm offers numerous deep learning training techniques, such as linguistic transition [60], spontaneous geometric transition [61], and spontaneous color dithering [62], to use on training images, yielding promising results. Images will be preprocessed and the YOLOv6 model’s training procedure will be improved in future work. Using transfer learning, YOLOv6’s generalization skills might be enhanced.

## 5. Limitations

**Figure 11:**
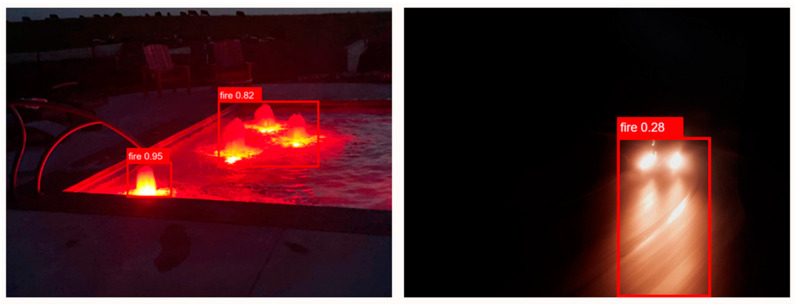
Limitation results for non-fire images at nighttime.

Figure 11 demonstrates that it is impossible to determine the quality of a model based on a single criterion rather than its entire performance. Our proposed model has significant drawbacks; for instance, when we tested the model in various situations, electric light or the sun in some cases were regarded as fire. To address this issue, we aim to improve the suggested model using additional datasets from other contexts [63,64,65,66,67]. In the custom dataset, we also did not add any classes for smoke. Therefore, if there is simply smoke present during the early fire stage, our model waits until it notices a fire. To improve our model and address the aforementioned problem, we are using large datasets, such as JFT-300M [68,69,70,71,72], which comprises 300 million labeled images.

## 6. Conclusions

In this study, we proposed an improved fast fire detection method to classify fires using a YOLOv6 network using deep learning approaches. Collecting sufficient image data for training models in forest fire detection is challenging, leading to data imbalance or overfitting concerns that impair the model’s effectiveness. One of the most efficacious methods of addressing overfitting is to increase the training dataset via data augmentation techniques. During the experiments, we found that image data augmentation approaches, such as geometric (affine) transformations, brightness/contrast enhancement, and data normalization, were effective in increasing the number of dataset images and improving the final accuracy rate. The effectiveness of DL models depends on the size and resolution of the training image datasets. Hence, it is important to extend the training dataset by data augmentation. The YOLOv6 model suggested in this research extracts features from the input picture using Efficient Net, which aids the model’s feature learning, boosts the network’s performance, and perfects the YOLOv3 model’s detection process for small targets. Preliminary findings from the experiments presented in this study demonstrate that the YOLOv6 model is superior to both the YOLOv3 and Faster R-CNN models. Real-time firing target detection is another capability of the YOLOv6 model. By detecting forest fires quickly and accurately, we can minimize the economic loss they cause, better safeguard forests and their biological surroundings, and promote sustainable growth of our resources.

Additionally, we noticed several restrictions in real-time applications, such as the inability to classify images containing smoke in our collection. Future research directions include improving the accuracy of the approach and addressing fuzzy situations in poorly illuminated environments. In the recognition and healthcare domains, we intend to create a compact model with reliable fire detection performance using 3D CNN/U-Net [73,74,75,76,77,78].

## Figures and Tables

**Table 1:** Allocation of fire images in the dataset.

Dataset	Open-Source Images	Video Frames	Total
Fire Images	2700	1300	4000

**Table 2:** Distribution of fire images in the dataset.

Dataset	Training Images	Testing Images	Total
Robmarkcole	1155	337	1492
Glenn–Jocher	1500	128	1628

**Table 3:** Allocation of fire and fire-like images in the dataset.

Dataset	Training Images	Testing Images	Total
Fire Images	7700	2300	11,000
Non-Fire Images	2300	0	2300

**Table 4:** The confusion matrix of the real and predicted categories for dichotomous problems.

Labeled Name	Predicted	Confusion Matrix
Positive	Positive	TP
Positive	Negative	FN
Negative	Positive	FP
Negative	Negative	TP

**Table 5:** Specifications of the hardware setup/configuration.

Hardware	Detailed Specifications
Graphic Processing Unit	GeForce RTX 2080 TI 11 GB (2 are installed)
Central Processing Unit	Intel Core 9 Gen i7-9700k (4.90 GHz)
Random Access Memory	DDR4 16 GB (4 are installed)
Storage	SSD: 512 GB HDD: 2 TB (2 are installed)
Motherboard	ASUS PRIME Z390-A
Operating System	Windows 10 Pro
Local Area Network	Internal port—10/100 Mbps External port—10/100 Mbps
Power	1000 W (+12 V Single Rail)

**Table 6:** Comparison of the proposed model with YOLOV3 and Faster R-CNN only on a small target firing dataset in regards to accuracy, recall, and mAP (mean Average Precision).

	Faster R-CNN	YOLOv3	YOLOv6
Precisions	29.83%	53.71%	93.48%
Recalls	15.70%	29.50%	28.29%
mAP	10.36%	28.10%	39.50%

## Data Availability

Not applicable.

## References

[1] Korean Statistical Information Service. http://kosis.kr.

[2] Ba R., Chen C., Yuan J., Song W., Lo S. (2019). SmokeNet: Satellite Smoke Scene Detection Using Convolutional Neural Network with Spatial and Channel-Wise Attention. Remote Sens..

[3] Dimitropoulos K., Barmpoutis P., Grammalidis N. (2015). Spatio-temporal flame modeling and dynamic texture analysis for automatic video-based fire detection. IEEE Trans. Circuits Syst. Video Technol..

[4] Valikhujaev Y., Abdusalomov A., Cho Y.I. (2020). Automatic Fire and Smoke Detection Method for Surveillance Systems Based on Dilated CNNs. Atmosphere.

[5] Barmpoutis P., Stathaki T., Dimitropoulos K., Grammalidis N. (2020). Early Fire Detection Based on Aerial 360-Degree Sensors, Deep Convolution Neural Networks and Exploitation of Fire Dynamic Textures. Remote Sens..

[6] Lu G., Gilabert G., Yan Y. (2005). Vision based monitoring and characterization of combustion flames. J. Phys. Conf. Ser..

[7] Gagliardi A., Saponara S. (2020). AdViSED: Advanced Video SmokE Detection for Real-Time Measurements in Antifire Indoor and Outdoor Systems. Energies.

[8] Toulouse T., Rossi L., Celik T., Akhloufi M. (2016). Automatic fire pixel detection using image processing: A comparative analysis of rule-based and machine learning-based methods. SIViP.

[9] Avgeris M., Spatharakis D., Dechouniotis D., Kalatzis N., Roussaki I., Papavassiliou S. (2019). Where There Is Fire There Is SMOKE: A Scalable Edge Computing Framework for Early Fire Detection. Sensors.

[10] Zhang Z., Zhao J., Zhang D., Qu C., Ke Y., Cai B. (2008). Contour based forest fire detection using FFT and wavelet. Proc. Int. Conf. CSSE.

[11] Celik T., Demirel H., Ozkaramanli H., Uyguroglu M. (2007). Fire detection using statistical color model in video sequences. J. Vis. Commun. Image Represent..

[12] Prema C.E., Vinsley S.S., Suresh S. (2018). Efficient flame detection based on static and dynamic texture analysis in forest fire detection. Fire Technol..

[13] Avazov K., Mukhiddinov M., Makhmudov F., Cho Y.I. (2022). Fire Detection Method in Smart City Environments Using a Deep-Learning-Based Approach. Electronics.

[14] Farkhod A., Abdusalomov A.B., Mukhiddinov M., Cho Y.-I. (2022). Development of Real-Time Landmark-Based Emotion Recognition CNN for Masked Faces. Sensors.

[15] Mamieva D., Abdusalomov A.B., Mukhiddinov M., Whangbo T.K. (2023). Improved Face Detection Method via Learning Small Faces on Hard Images Based on a Deep Learning Approach. Sensors.

[16] Abdusalomov A.B., Safarov F., Rakhimov M., Turaev B., Whangbo T.K. (2022). Improved Feature Parameter Extraction from Speech Signals Using Machine Learning Algorithm. Sensors.

[17] Khan F., Tarimer I., Alwageed H.S., Karadağ B.C., Fayaz M., Abdusalomov A.B., Cho Y.-I. (2022). Effect of Feature Selection on the Accuracy of Music Popularity Classification Using Machine Learning Algorithms. Electronics.

[18] Luo Y., Zhao L., Liu P., Huang D. (2018). Fire smoke detection algorithm based on motion characteristic and convolutional neural networks. Multimed. Tools Appl..

[19] Sharma J., Granmo O.C., Goodwin M., Fujita H., Selamat A., Lin J.C.W., Ali M. (2021). Emergency Analysis: Multitask Learning with Deep Convolutional Neural Networks for Fire Emergency Scene Parsing. Advances and Trends in Artificial Intelligence. Artificial Intelligence Practices.

[20] Li P., Zhao W. (2020). Image fire detection algorithms based on convolutional neural networks. Case Stud. Therm. Eng..

[21] Muhammad K., Ahmad J., Mehmood I., Rho S., Baik S.W. (2018). Convolutional Neural Networks Based Fire Detection in Surveillance Videos. IEEE Access.

[22] Pan H., Badawi D., Cetin A.E. (2020). Computationally Efficient Wildfire Detection Method Using a Deep Convolutional Network Pruned via Fourier Analysis. Sensors.

[23] Li T., Zhao E., Zhang J., Hu C. (2019). Detection of Wildfire Smoke Images Based on a Densely Dilated Convolutional Network. Electronics.

[24] Kim B., Lee J. (2019). A Video-Based Fire Detection Using Deep Learning Models. Appl. Sci..

[25] Joseph R., Divvala S., Girshick R., Farhadi A. You only look once: Unified, real-time object detection. Proceedings of the IEEE Conference on Computer Vision and Pattern Recognition.

[26] Park M., Ko B.C. (2020). Two-Step Real-Time Night-Time Fire Detection in an Urban Environment Using Static ELASTIC-YOLOv3 and Temporal Fire-Tube. Sensors.

[27] Abdusalomov A., Baratov N., Kutlimuratov A., Whangbo T.K. (2021). An improvement of the fire detection and classification method using YOLOv3 for surveillance systems. Sensors.

[28] Mukhiddinov M., Abdusalomov A.B., Cho J. (2022). Automatic Fire Detection and Notification System Based on Improved YOLOv4 for the Blind and Visually Impaired. Sensors.

[29] Mukhiddinov M., Abdusalomov A.B., Cho J. (2022). A Wildfire Smoke Detection System Using Unmanned Aerial Vehicle Images Based on the Optimized YOLOv5. Sensors.

[30] Abdusalomov A.B., Mukhiddinov M., Kutlimuratov A., Whangbo T.K. (2022). Improved Real-Time Fire Warning System Based on Advanced Technologies for Visually Impaired People. Sensors.

[31] Robmarkcole 2022, Fire-Detection-from-Images, Github. https://github.com/robmarkcole/fire-detection-from-images.

[32] Glenn Jocher 2022, Yolov5, Github. https://github.com/ultralytics/yolov5.

[33] Kuldoshbay A., Abdusalomov A., Mukhiddinov M., Baratov N., Makhmudov F., Cho Y.I. (2022). An improvement for the automatic classification method for ultrasound images used on CNN. Int. J. Wavelets Multiresolution Inf. Process..

[34] Safarov F., Temurbek K., Jamoljon D., Temur O., Chedjou J.C., Abdusalomov A.B., Cho Y.-I. (2022). Improved Agricultural Field Segmentation in Satellite Imagery Using TL-ResUNet Architecture. Sensors.

[35] Avazov K., Hyun A.E., S Sami A.A., Khaitov A., Abdusalomov A.B., Cho Y.I. (2023). Forest Fire Detection and Notification Method Based on AI and IoT Approaches. Future Internet.

[36] Abdusalomov A.B., Islam B.M.S., Nasimov R., Mukhiddinov M., Whangbo T.K. (2023). An Improved Forest Fire Detection Method Based on the Detectron2 Model and a Deep Learning Approach. Sensors.

[37] Redmon J. Darknet: Open-Source Neural Networks in C. 2013–2016. http://pjreddie.com/darknet/.

[38] Bochkovskiy A., Wang C.Y., Liao H.Y.M. (2020). YOLOv4, Optimal Speed and Accuracy of Object Detection. arXiv.

[39] Li C., Li L., Jiang H., Weng K., Geng Y., Li L., Ke Z., Li Q., Cheng M., Nie W. (2022). YOLOv6: A single-stage object detection framework for industrial applications. arXiv.

[40] Sovit Rath YOLOv6 Object Detection–Paper Explanation and Inference. https://learnopencv.com/yolov6-object-detection/#disqus_thread.

[41] YOLOv6: A Single-Stage Object Detection Framework for Industrial Applications. https://github.com/meituan/YOLOv6..

[42] Robert Singh A., Athisayamani S., Sankara Narayanan S., Dhanasekaran S. (2021). Fire Detection by Parallel Classification of Fire and Smoke Using Convolutional Neural Network. Computational Vision and Bio-Inspired Computing.

[43] Wang Z., Zhang H., Hou M., Shu X., Wu J., Zhang X., Li K., Coombs T., He J., Tian Y., Niu Q., Yang Z. (2021). A Study on Forest Flame Recognition of UAV Based on YOLO-V3 Improved Algorithm. Recent Advances in Sustainable Energy and Intelligent Systems (LSMS 2021, ICSEE 2021).

[44] Hou F., Zhang Y., Fu X., Jiao L., Zheng W. (2021). The Prediction of Multistep Traffic Flow Based on AST-GCN-LSTM. J. Adv. Transp..

[45] Zhang Y., Ren J., Wang R., Fang F., Zheng W. (2021). Multi-Step Sequence Flood Forecasting Based on MSBP Model. Water.

[46] Tan M., Le Q.V. EfficientNet: Rethinking model scaling for convolutional neural networks. Proceedings of the International Conference on Machine Learning (ICML).

[47] Sandler M., Howard A., Zhu M., Zhmoginov A., Chen L.-C. (2018). Inverted Residuals and Linear Bottlenecks: Mobile Networks for Classification, Detection and Segmentation. arXiv.

[48] Safarov F., Kutlimuratov A., Abdusalomov A.B., Nasimov R., Cho Y.-I. (2023). Deep Learning Recommendations of E-Education Based on Clustering and Sequence. Electronics.

[49] Abdusalomov A., Whangbo T.K. (2017). An improvement for the foreground recognition method using shadow removal technique for indoor environments. Int. J. Wavelets Multiresolut. Inf. Process..

[50] Abdusalomov A., Whangbo T.K. (2019). Detection and Removal of Moving Object Shadows Using Geometry and Color Information for Indoor Video Streams. Appl. Sci..

[51] Abdusalomov A., Mukhiddinov M., Djuraev O., Khamdamov U., Whangbo T.K. (2020). Automatic salient object extraction based on locally adaptive thresholding to generate tactile graphics. Appl. Sci..

[52] Kutlimuratov A., Abdusalomov A., Whangbo T.K. (2020). Evolving Hierarchical and Tag Information via the Deeply Enhanced Weighted Non-Negative Matrix Factorization of Rating Predictions. Symmetry.

[53] Kutlimuratov A., Abdusalomov A.B., Oteniyazov R., Mirzakhalilov S., Whangbo T.K. (2022). Modeling and Applying Implicit Dormant Features for Recommendation via Clustering and Deep Factorization. Sensors.

[54] Farkhod A., Abdusalomov A., Makhmudov F., Cho Y.I. (2021). LDA-Based Topic Modeling Sentiment Analysis Using Topic/Document/Sentence (TDS). Model. Appl. Sci..

[55] Akmalbek A., Djurayev A. (2016). Robust shadow removal technique for improving image enhancement based on segmentation method. IOSR J. Electron. Commun. Eng..

[56] Abdusalomov A., Whangbo T.K., Djuraev O. (2016). A Review on various widely used shadow detection methods to identify a shadow from images. Int. J. Sci. Res. Publ..

[57] Mukhamadiyev A., Mukhiddinov M., Khujayarov I., Ochilov M., Cho J. (2023). Development of Language Models for Continuous Uzbek Speech Recognition System. Sensors.

[58] Jie H., Li S., Gang S., Wu E. Squeeze-and-Excitation Networks. Proceedings of the IEEE Conference on Computer Vision and Pattern Recognition (CVPR), Salt Lake City.

[59] Howard A.G., Zhu M., Chen B., Kalenichenko D., Wang W., Wey T., Andreetto M., Adam H. (2017). Mobilenets: Efficient convolutional neural networks for mobile vision applications. arXiv.

[60] Zhao C., Zheng W. Fast Traffic Sign Recognition Algorithm Based on Multi-scale Convolutional Neural Network. Proceedings of the 2020 Eighth International Conference on Advanced Cloud and Big Data (CBD).

[61] Wang R., Fang F., Cui J., Zheng W. (2022). Learning self-driven collective dynamics with graph networks. Sci. Rep..

[62] Zheng W., Zhang S., Xu N. (2018). Jamming of packings of frictionless particles with and without shear. Chin. Phys. B.

[63] Zhang X., Qian K., Jing K., Yang J., Yu H. Fire Detection based on Convolutional Neural Networks with Channel Attention. Proceedings of the 2020 Chinese Automation Congress (CAC).

[64] Saponara S., Elhanashi A., Gagliardi A. (2021). Real-time video fire/smoke detection based on CNN in antifire surveillance systems. J. Real-Time Image Proc..

[65] Li W., Yu Z. A Lightweight Convolutional Neural Network Flame Detection Algorithm. Proceedings of the 2021 IEEE 11th International Conference on Electronics Information and Emergency Communication (ICEIEC).

[66] Avazov K., Abdusalomov A., Cho Y.I. (2020). Automatic moving shadow detection and removal method for smart city environments. J. Korean Inst. Intell. Syst..

[67] Turimov Mustapoevich D., Muhamediyeva Tulkunovna D., Safarova Ulmasovna L., Primova H., Kim W. (2023). Improved Cattle Disease Diagnosis Based on Fuzzy Logic Algorithms. Sensors.

[68] Sun C., Shrivastava A., Singh S., Gupta A. Revisiting Unreasonable Effectiveness of Data in Deep Learning Era. Proceedings of the IEEE International Conference on Computer Vision (ICCV).

[69] Mukhiddinov M., Muminov A., Cho J. (2022). Improved Classification Approach for Fruits and Vegetables Freshness Based on Deep Learning. Sensors.

[70] Luo D., Wang D., Guo H., Zhao X., Gong M., Ye L. Detection method of tubular target leakage based on deep learning. Proceedings of the Seventh Symposium on Novel Photoelectronic Detection Technology and Application.

[71] Mumuni A., Mumuni F. (2021). CNN architectures for geometric transformation-invariant feature representation in computer vision: A review. SN Comput. Sci..

[72] Kayhan O.S., Gemert J.C. On translation invariance in cnns: Convolutional layers can exploit absolute spatial location. Proceedings of the IEEE/CVF Conference on Computer Vision and Pattern Recognition.

[73] Nodirov J., Abdusalomov A.B., Whangbo T.K. (2022). Attention 3D U-Net with Multiple Skip Connections for Segmentation of Brain Tumor Images. Sensors.

[74] Jakhongir N., Abdusalomov A., Whangbo T.K. 3D Volume Reconstruction from MRI Slices based on VTK. Proceedings of the 2021 International Conference on Information and Communication Technology Convergence (ICTC).

[75] Ayvaz U., Gürüler H., Khan F., Ahmed N., Whangbo T., Abdusalomov A. (2022). Automatic Speaker Recognition Using Mel-Frequency Cepstral Coefficients through Machine Learning. CMC-Comput. Mater. Contin..

[76] Makhmudov F., Mukhiddinov M., Abdusalomov A., Avazov K., Khamdamov U., Cho Y.I. (2020). Improvement of the end-to-end scene text recognition method for “text-to-speech” conversion. Int. J. Wavelets Multiresolut. Inf. Process..

[77] Wafa R., Khan M.Q., Malik F., Abdusalomov A.B., Cho Y.I., Odarchenko R. (2022). The Impact of Agile Methodology on Project Success, with a Moderating Role of Person’s Job Fit in the IT Industry of Pakistan. Appl. Sci..

[78] Umirzakova S., Abdusalomov A., Whangbo T.K. Fully Automatic Stroke Symptom Detection Method Based on Facial Features and Moving Hand Differences. Proceedings of the 2019 International Symposium on Multimedia and Communication Technology (ISMAC).

